# Costs and cost-effectiveness of integrated screening for non-communicable diseases in TB contacts

**DOI:** 10.5588/ijtldopen.24.0625

**Published:** 2025-03-12

**Authors:** Y. Hamada, R. Mukora, R. Pelusa, T. Ntshiqa, J. Shedrawy, K. Velen, I. Abubakar, S. Charalambous, S. Walker, M.X. Rangaka

**Affiliations:** ^1^ Institute for Global Health, University College London, London, UK;; ^2^ Aurum Institute, Johannesburg, South Africa;; ^3^ Department of Global Public Health, WHO Collaboration Centre On Tuberculosis and Social Medicine, Karolinska Institute, Stockholm, Sweden;; ^4^ School of Public Health, Wits University, Johannesburg, South Africa;; ^5^ Centre for Health Economics, University of York, York, UK;; ^6^ School of Public Health, and Clinical Infectious Disease Research Institute-Africa, University of Cape Town, Cape Town, South Africa.

**Keywords:** economic analysis, integration, chronic diseases, contact investigation, tuberculosis

## Abstract

**BACKGROUND:**

Integrating non-communicable disease (NCD) screening into TB household contact investigations may identify undiagnosed NCDs and reduce the burden of both conditions. However, evidence on the costs and cost-effectiveness of this approach is limited.

**METHOD:**

We conducted a cross-sectional study in South Africa to assess patient and provider costs for NCD screening (hypertension, diabetes, chronic kidney disease, dyslipidaemia). Incremental costs per NCD case identified were calculated. Using a decision tree model, we estimated incremental costs per disability-adjusted life year (DALY) averted over 10 years from a healthcare perspective, with cardiovascular disease (CVD) risk estimated using the WHO prediction model.

**RESULTS:**

The incremental cost was USD72.3 per contact screened and USD334.0 per NCD case identified. Integrated screening reduced mean 10-year CVD risk from 5.7% to 2.7% among contacts with NCDs. The incremental cost-effectiveness ratio (ICER) was USD27,043.6 per DALY averted, exceeding South Africa’s threshold of USD3,708. Management of identified NCDs, mainly drug costs, comprised over 80% of total incremental costs. The ICER decreased in populations with a high risk for NCDs.

**CONCLUSION:**

Integrated NCD screening was not cost-effective, mainly due to subsequent care costs. Prioritising individuals at high risk for NCDs can improve cost-effectiveness.

TB imposes a considerable financial strain on affected individuals and their households. National surveys in 37 low- and middle-income countries found that 13–92% of TB-affected households face catastrophic costs.^1^ Additionally, low- and middle-income countries are facing a growing non-communicable disease (NCD) burden, which can compound the economic challenges in TB-affected households.^2,3^

Our research revealed a high prevalence of undiagnosed NCDs, particularly diabetes and hypertension, among household contacts of individuals with TB.^4,5^ Smoking, a major risk factor for NCDs, also tends to be more prevalent in these households.^6^ While expanding NCD prevention and control measures may initially increase health system costs, the WHO projects significant long-term benefits. Interventions for NCDs are estimated to save approximately 7 million lives and prevent 10 million heart disease and stroke cases, resulting in a return of USD7 for every dollar spent on NCD interventions.^7^

The WHO recommends ‘best buy’ interventions for NCDs.^8^ Among these interventions is the management of cardiovascular disease (CVD) risk in individuals with a high 10-year risk (≥30% or ≥20%, depending on resource availability), which includes treating hypertension and diabetes and using statins. Given the significant burden of undiagnosed hypertension and diabetes among household contacts of TB patients, incorporating NCD screening into household contact investigations could be a valuable strategy for managing CVD risk. This approach can enhance NCD control by utilising existing infrastructure and resources.

However, there is limited data on the costs and cost-effectiveness of integrating NCD screening into TB contact investigations. Only one study, conducted in Myanmar, has evaluated the cost-effectiveness of diabetes screening within TB contact tracing.^9^ However, that study only considered the costs associated with the initial two visits and did not account for the ongoing management of diabetes or other NCDs. To address this gap, we estimated the costs and cost-effectiveness of integrating NCD screening within household contact investigations in South Africa.

## METHOD

### Design

We collected data on the costs of NCD screening (hypertension, diabetes, chronic kidney diseases [CKD] and dyslipidaemia) from a pilot study in Ekurhuleni, South Africa. Study details are reported elsewhere.^10^

Incremental screening costs per NCD detected were estimated for providers and patients, focusing on costs up to the point of referral, such as travel and consultation fees, while excluding treatment costs.

Additionally, we employed a decision tree model analysis to estimate incremental cost per disability-adjusted life year (DALY) averted over 10 years from a healthcare perspective. This approach was modelled similarly to that used by Sando et al.^11^ Due to insufficient data, we did not incorporate a societal perspective.^12^

We obtained written informed consent from all study participants. Ethical approval was obtained from the Ethics Committee at the University of the Witwatersrand, Johannesburg, South Africa (Ref: 220210) and University College London, London, UK (Ref:21569/002).

### Intervention description

In the integrated screening, a study nurse screened household contacts of individuals with TB for hypertension, diabetes, and CKD. Diabetes was defined as glycated haemoglobin (HbA1c) ≥6.5%, hypertension as systolic blood pressure ≥140 mmHg and/or diastolic blood pressure ≥90 mmHg, and CKD as an estimated glomerular filtration rate (eGFR) <60 mL/min.^13–15^ Total cholesterol was also measured to estimate CVD risk (see below). Contacts newly diagnosed with NCDs were referred to a nearby clinic for further management.

We assumed that referred contacts received treatment according to the South African PC 101 guideline, as in Basu et al.'s cost-effectiveness analysis.^16,17^ Table 1 summarises treatment assumptions.

**Table 1.** Treatment algorithms.^*^

Hypertension	
SBP 140–149 mmHg *OR* (SBP <140 mmHg and DBP ≥90 mmHg)	Diuretics
SBP 150–159 mmHg	Diuretics + ACEI
SBP 160–169 mmHg	Diuretics + ACEI + Ca-blocker
≥SBP 170 mmHg	Diuretics + ACEI + Ca-blocker + beta-blocker
Diabetes	
HbA1c 6.5–8.5%	Metformin
HbA1c 8.5–10%	Metformin + sulfonylurea glibenclamide
HbA1c >10%	Metformin + sulfonylurea glibenclamide + insulin, basal
Statin	
History of cardiovascular disease *OR* 10-year cardiovascular disease risk >20% *OR* diabetic with hypertension, obesity, smoking, *OR* age ≥40 years	Statin

* We presumed that, based on initial blood pressure and HbA1c levels, contacts would immediately commence a comprehensive treatment regimen aimed at achieving target levels (SBP <140 mmHg and HbA1c <7.0%), without gradual titration. For isolated diastolic hypertension, we assumed that contacts would be prescribed only the first-line medication.

SBP = systolic blood pressure; DBP = diastolic blood pressure; ACEI = angiotensin-converting enzyme inhibitor; Ca = calcium; HbA1c = glycated haemoglobin.

CVD risk over 10 years was estimated using the WHO risk prediction model, incorporating age, sex, smoking status, presence of diabetes, systolic blood pressure, and total cholesterol levels.^18^ Effectiveness parameters were drawn from Basu et al. and Kasaie et al. (Supplementary Table S1).^16,19^

### Estimating costs

As part of the cross-sectional study, we interviewed research staff to assess the additional time required for NCD screening alongside household TB investigations. This time was combined with their hourly wages to estimate human resource costs. We extracted costs related to laboratory tests, equipment (e.g., blood pressure monitors), and training from financial records.

To capture direct and indirect costs incurred by contacts, we administered a questionnaire to study participants diagnosed with NCDs (see Supplementary Data 2). All costs were converted from ZAR to USD using the 2022 World Bank exchange rate.

For the decision tree analysis, beyond the initial costs of integrated screening, we included the ongoing costs for managing NCDs (e.g. medication and consultation fees) and acute care costs for CVD events, using estimates from Basu et al. (Supplementary Table S2).^16^ Costs were discounted at 3% per year.^20^ In the base case, we assumed that contacts would start treatment for their underlying NCD once they developed CVD at Year 5; otherwise, no treatment was initiated.

### Outcome

We adapted the approach used by Sando et al.^11^ to estimate CVD risk, costs, and effects for contacts newly diagnosed with NCDs in the intervention group and for those not diagnosed in the base case. We calculated the individual 10-year CVD risk using the WHO risk prediction model.^18^ Based on the 2021 Global Burden of Disease (GBD) estimate for South Africa, we assumed that 72% of CVD events were ischemic heart disease and 28% were strokes.^21^ Other outcomes (e.g. diabetic retinopathy and renal failure) were not considered due to the lack of relevant variables to estimate their risk.

For each CVD event, we calculated years of life lost (YLL) and years lived with disability (YLD) using case fatality ratios derived from the 2019 GBD study.^21^ We assumed that CVD events would occur at year 5, and YLLs and YLDs were estimated using age- and sex-specific life expectancies for up to 5 years following the event.^22^ For disutility weights post non-fatal CVD event, we adopted the weight used by Basu et al.^16^

Supplementary Table S3 summarises the parameters used to calculate DALYs. We calculated DALYs for each approach (screening vs. no screening) by summing YLLs and YLDs, with a 3% annual discount rate.

### Analysis

#### Incremental costs per NCD identified

First, we calculated incremental costs for providers, patients, and total costs. These costs included those from household screenings and initial clinic visits to investigate contacts diagnosed with NCDs, excluding downstream NCD treatment costs.

Indirect costs, such as income loss for contacts and their attendants due to the initial referral, were estimated in two ways. First, we considered self-reported income loss. However, relying on self-reported income underestimates the productivity loss of non-waged workers.^23^ Thus, we also used the ‘minimum wage approach’, multiplying clinic and travel time by the minimum hourly wage in 2022 (USD1.42).

We calculated the incremental costs per new NCD case identified. In the base case, no NCD cases were assumed to be identified.

### Incremental costs and DALYs averted over 10 years

We assessed the potential costs and DALYs averted by integrating NCD screening and subsequent treatment for contacts newly diagnosed with an NCD, compared to no screening and no treatment, using a decision tree model (Figure) over a 10-year period from the healthcare perspective. The incremental cost-effectiveness ratio (ICER) was calculated as the incremental cost per DALY averted.

**Figure.**
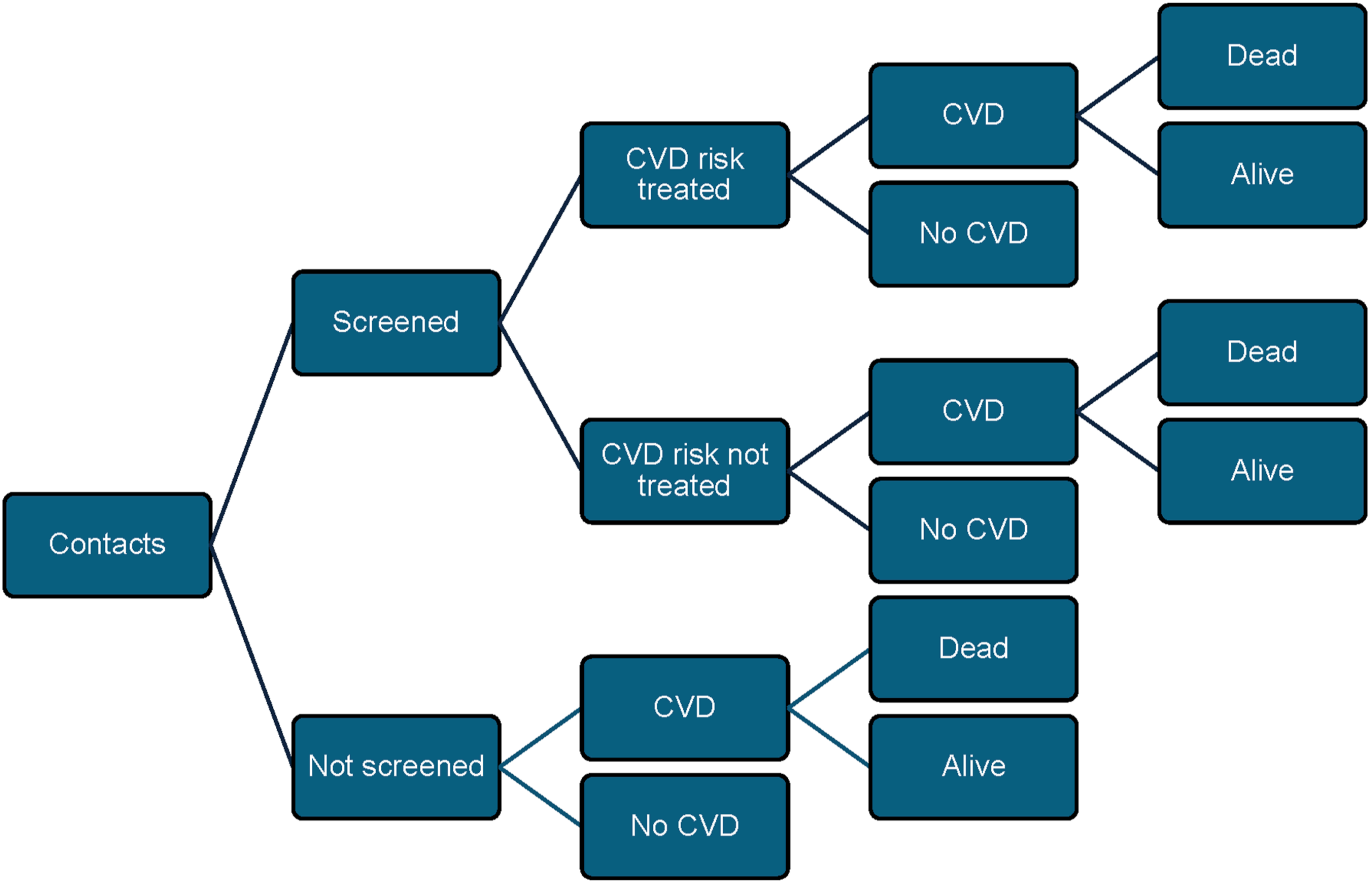
Decision tree. CVD = cardiovascular disease; NCD = non-communicable disease.

Following WHO guidance,^18^ which recommends CVD risk assessment and diabetes testing in high-risk individuals, we evaluated three different strategies targeting specific groups. The first strategy provided CVD risk assessment, blood pressure measurement, and diabetes testing to all adults over 40. The second and third strategies provided CVD risk and blood pressure assessments to all adults over 40 and younger adults with risk factors, with diabetes testing limited to those over 40 who were overweight (second strategy) or obese (third strategy) (see Supplementary Table S4)

### Sensitivity analysis

We conducted a probabilistic sensitivity analysis to assess the impact of parameter uncertainty. Statistical distributions were assigned to key parameters (e.g., disutility, systolic blood pressure reduction, relative risks), and random samples were drawn. We resampled the study cohort with replacement and repeated the calculation of incremental costs and DALYs averted 10,000 times. The net health benefit was calculated with uncertainty intervals (2.5^th^ and 97.5^th^ percentiles). We applied the South African cost-effectiveness threshold of USD3,015 per DALY from Edoka et al.,^24^ adjusted to USD3,708 for 2022 using a 3% annual inflation rate.

We conducted a scenario analysis to assess how varying 10-year CVD risk levels impacted cost-effectiveness. The baseline CVD risk before treatment was systematically increased from 1.0 to 5.0 in 0.1 increments, accounting for parameter uncertainty. We plotted the probability of cost-effectiveness (ICER < USD3,708) against the median CVD risk.

## RESULTS

We enrolled and screened 291 participants in the study, 63 of whom were diagnosed with an NCD and referred to a clinic. Of these, 54 followed through with the clinic visit, and 44 participated in a survey to collect data on screening costs. Characteristics of participants are presented in Supplementary Table S5

### Costs for integrated NCD screening

All but one participant who attended a clinic visited a public clinic, and none of those who visited a public clinic paid any registration fees. Supplementary Table S6 presents the detailed results of the cost survey. The total patient costs, including both direct and indirect expenses, were calculated at USD2.93 based on self-reported income loss (Supplementary Table S7). This figure increased to USD5.14 when calculated using the minimum wage approach.

The NCD screening added an average of 19 extra minutes of staff time, leading to an additional cost of USD9.79 per contact. The total provider cost per contact screened was USD71.60, with direct costs for laboratory tests and consumables comprising most of this amount (USD60.34) (Supplementary Table S8). Among these, HbA1c tests represented the largest portion, accounting for 38.9% of the costs.

Overall, the total incremental cost for NCD screening was USD72.30 per contact screened (Table 2). The incremental cost per case of at least one NCD identified was USD334.00, with most of this cost (USD331.50 or 99.3%) attributed to provider costs. The total cost per NCD identified decreased to USD265.3 when laboratory tests were limited to HbA1c and total cholesterol, with a further reduction to USD199.0 when restricted to contacts aged ≥40 years.

**Table 2.** Summary of incremental costs for NCD screening.

	Incremental cost per person screened (USD)	Incremental cost per NCD identified (USD)
Provider cost per person screened	71.6	331.5
Patient cost per person screened	0.72	2.5
Total cost per person screened	72.3	334.0

NCD = non-communicable disease; USD = US dollar.

### Cost-effectiveness of integrated NCD screening over 10 years

With the intervention, the median 10-year CVD risk decreased from 5.7% (interquartile range [IQR] 1.8–12.3) to 2.7% (IQR 1.0–5.1) (Table 3). As a result, DALYs were reduced from 3.7 years per 100 persons to 1.8 years.

**Table 3.** Incremental cost, DALYs averted and cost-effectiveness of integrated NCD screening within contact investigation.

	Intervention (integrated NCD screening)	Status quo
10-year CVD risk in contacts found to have NCD, %, median [IQR]	2.7 [1.0–5.1]	5.7 [1.8–12.3]
YLL/100 persons	1.2	2.5
YLD/100 persons	0.8	1.7
DALYs (discounted)/100 persons	1.7	3.5
Incremental cost for screening/contact screened, USD	71.6	—
Cost for subsequent management/contact screened, USD	442.6	26.4
Incremental cost/contact screened	487.9	—
Incremental cost/DALY averted, USD	27043.6	—

DALY = disability-adjusted life-year; NCD = non-communicable disease; CVD = cardiovascular disease; IQR = interquartile range; YLL = years of life lost; YLD = years lived with disability; USD = US dollar.

The incremental cost for NCD screening and associated treatment averaged USD487.9 per contact screened, with 85% of this amount (USD416.20) attributed to the costs of management following the screening. Medications accounted for 66% of those costs. The ICER was USD27,043.6 per DALY averted. When the screening costs were excluded, the ICER was USD23,073.7, exceeding the cost-effectiveness threshold.

Across three strategies targeting different sub-groups, the lowest ICER was observed when screening was limited to individuals over 40 years old, at USD20,551.3 per DALY averted (Supplementary Table S9). Supplementary Table S10 and Supplementary Figure S1 present the sensitivity analysis results, which indicate that net health benefits were negative across all scenarios, suggesting an overall loss in health benefits. The upper limit of the uncertainty intervals (i.e., 97.5^th^ percentiles) was largest at –19.9 when NCD screening was limited to individuals over 40 years old.

Supplementary Figure S2 illustrates the changes in the probability of the intervention being cost-effective as the 10-year CVD risk among contacts increased. When the median risk reached 20%, the likelihood of the intervention being cost-effective began to rise sharply. At a median 10-year CVD risk of 27%, the probability of it being cost-effective was 51%.

## DISCUSSION

To our knowledge, this is the first study to evaluate the incremental costs and cost-effectiveness of integrated NCD screening within household contact investigations for TB. The study found an ICER of USD27,043 per DALY averted, exceeding South Africa’s cost-effectiveness threshold.^24^ The costs for managing NCDs identified through screening accounted for over 80% of incremental costs. These findings suggest that the cost-effectiveness of integrated NCD screening largely depends on the cost-effectiveness of subsequent care, which was not cost-effective. Sensitivity analyses suggest potential improvements in cost-effectiveness by optimising test selection and target populations.

In contrast, Basu et al. demonstrated that scaling up CVD risk management in the South African general population could be cost-saving.^16^ Their analysis considered additional outcomes, such as renal failure, congestive heart failure linked to hypertension, and microvascular complications of diabetes. The improvement of glycaemic control might reduce the risk of the development of TB or other infectious diseases.^25,26^ Our study, constrained by available data, did not account for these broader outcomes. Moreover, we did not account for recurrent CVD events, likely underestimating cost-effectiveness.

Another key difference between the studies is the baseline CVD risk. In Basu et al., the 10-year CVD risk was 9.9%, nearly double the 5.7% observed in our cohort, likely contributing to their more favourable cost-effectiveness results. Our analysis showed a lower ICER when screening was restricted to adults over 40, a higher-risk group, highlighting the benefits of focusing NCD screening on high-risk populations.

Additionally, South Africa’s National Strategic Plan for NCDs (2022–2027) aims for ‘90% of all individuals over 18 to know their blood pressure and blood glucose status’.^27^ Under this framework, assuming no treatment for hypertension or diabetes in the baseline scenario may be unrealistic. A more accurate comparison might involve evaluating alternative screening strategies rather than assuming the absence of screening and treatment for these conditions.

Integrating NCD screening increased provider costs by USD71.60 per contact, with 84% of the costs due to laboratory tests and consumables. HbA1c testing had the highest unit cost (USD23.47), followed by total cholesterol (USD7.82), serum creatinine (USD6.85), and blood glucose (USD6.85). Scaling up screening with bulk purchasing could reduce unit costs for tests, kit assembly, and sample transportation.

No diabetes cases were diagnosed based solely on random blood glucose, and 85% of CKD cases occurred in participants with comorbidities like diabetes, hypertension, or HIV. Limiting diabetes screening to HbA1c and targeting serum creatinine testing to those with comorbidities could further reduce costs.

This study has several limitations. First, societal costs, such as productivity losses from NCDs, were excluded, and their inclusion might have improved cost-effectiveness outcomes. Patient costs (e.g., clinic waiting time, travel, and out-of-pocket expenses) were not included, though initial referral data suggested these were not substantial. Second, potential losses along the care cascade were not accounted for, while suboptimal treatment uptake and retention could diminish screening effectiveness. Third, the study assumed that integrating NCD screening would not impact TB care. However, such integration could strain healthcare workers, limiting household visits or linkage to TB care. Conversely, integration might bring benefits, as seen in integrating HIV care with other health services.^27^

## CONCLUSION

In summary, this study was unable to confirm the cost-effectiveness of integrating NCD screening into household contact investigations. This outcome may be influenced by methodological constraints inherent to our economic evaluation. It is also essential to interpret these results within the context of South Africa’s national plan to expand NCD management. The findings indicate that tailoring screening to specific NCDs and targeting high-risk groups may enhance cost-effectiveness. Future studies on cost-effectiveness should utilise empirical data, ideally derived from effectiveness trials.

## Supplementary Material


