# Supplementary material for: Costs and cost-effectiveness of integrated screening for non-communicable diseases in TB contacts
Source: IJTLD Open. 2025 Mar 12;2(3):160–5. doi: 10.5588/ijtldopen.24.0625 (PMC11906026; doi:10.5588/ijtldopen.24.0625)
Supplement: Supplementary file 1 [file ijtldopen24-0625_supplementarydata1.pdf]

http://dx.doi.org/10.5588/ijtldopen.24.0625

Costs and cost-effectiveness of integrated screening for non-communicable diseases in TB contacts

Supplementary materials

Appendix 1. Supplementary tables and figures

Table A1. Effectiveness of interventions

**SBP reduction**	Reduction in systolic blood pressure (mmHg, 95% CI)	Source
ACE inhibitor	8.5 (7.9, 9.0)	Law, et al. cited by Basu et al.
Beta-blocker	9.2 (8.6, 9.9)	
Thiazide diuretic	8.8 (8.3, 9.4)	
Calcium channel blocker	8.8 (8.3, 9.2)	
**Relative risk reduction**		
Relative risk for atherosclerotic cardiovascular disease events according to s a function of age (in years) and change in systolic blood pressure (ΔSBP)	$RR = 2^{\Delta SBP(-0.0000184775\times age2+0.001584\times age+0.028672)}$	Smith-Spangler, et al. cited by Basu et al.
Risk for CVD in people treated for diabetes	RR= 0.79 (95% CI 0.64-0.98)	Lamanna, et al. cited by Kasaie, et al.
Relative risk for CVD in people given statin therapy	RR= 0.79 (95% CI 0.77-0.81)	Cholesterol Treatment Trialists' Collaboration, 2015. cited by Basu et al.

ACE: Angiotensin-converting enzyme; SBP: systolic blood pressure; RR: relative risk; CVD: cardiovascular disease; ACE: Angiotensin-converting enzyme inhibitor

Note: When multiple interventions are given, the overall reduction in risk was assumed to be multiplicative. We did not account for a potential reduction in CVD risk through changes in HbA1c levels because the cardiovascular benefits of other diabetes therapies are less definitive, and the WHO model for predicting CVD risk does not incorporate baseline HbA1c levels.

Table A2. Costs for the management of NCD

Condition	Item	Cost (US$)
Hypertension	Annual physician visit*	20/yr
	Nurse visit every three months	37/yr
	Annual electrolytes and urea labs	9/yr
	Thiazide	52/yr
	ACE inhibitor	69/yr
	Calcium channel blocker	34/yr
	Beta-blocker	36/yr
Dyslipidaemia	Annual physician visit	20/yr
	Statin	34/yr
Type 2 diabetes mellitus	Annual physician visit	20/yr
	Nurse visits every three months	37/yr
	Hemoglobin A1c every 6 months	13/yr
	Annual electrolytes and urea labs	9/yr
	Metformin	62/yr
	Sulfonylurea	204/yr
	Insulin, basal	115/yr
Ischemic heart disease	Acute care for IHD	1089 (once)
	Monthly nurse visits for 6 months	55
	annual physician follow-up	20/yr
	Asprin	1/yr
	Beta-blocker	36/yr
	Statin	34/yr
	ACE inhibitor	125/yr
	IHD – electrolytes and urea every 6 months	17/yr
Stroke	Acute care for stroke	2202 (once)
	Monthly nurse visits for 6 months post-stroke	55/yr
	Stroke – aspirin 1/yr	1/yr
	Stroke – statin	34/yr
	Annual physician follow-up	20/yr

IHD: ischemic heart disease; ACE: Angiotensin-converting enzyme

Note: Basu et al. estimated costs for the treatment of each NCD by breaking them down into care components as per the standard guidelines. They then extracted costs for these from national data sources, including the South African Uniform Patient Fee schedule and the National Health Laboratory Service fees. For contacts with multiple concurrent risk factors or conditions, the cost of annual physician visits, nurse visits, other services, and overlapping laboratory tests or medications were counted only once.

Table A3. Parameters used to calculate DALYs

		Source
Risk for CVD	Prediction model over 10 years (72% of CVD events are assumed to be IHD, based on GBD 2021)	WHO risk prediction model
Case fatality due to IHD	17.6%	GBD 2021 (approximated by deaths/incidence)
Case fatality due to Stroke	57.4%	GBD 2021 (approximated by deaths/incidence)
Disutility due to CVD	0.28 (0.06, 0.57)	Basu, 2018

IHD: ischemic heart disease.

Table A4. Targeted strategies focused on groups at risk for cardiovascular diseases

Strategy 1	All adults > 40 year
Strategy 2	• CVD risk assessment and blood pressure measurement:<br>○ Adults aged > 40 years;<br>○ Current smokers; or<br>○ People who are overweight<br>• Testing for diabetes<br>○ Adults aged > 40 years who are overweight
Strategy 3	• CVD risk assessment and blood pressure measurement:<br>○ Adults aged > 40 years;<br>○ Current smokers; or<br>○ People who are overweight<br>• Testing for diabetes<br>○ Adults aged > 40 years who are obese

CVD: cardiovascular diseases

Table A5. Characteristics of participants who participated in the cost survey

Variables	Participants (N = 44)
Age (median [IQR])	56.50 [42.00, 67.00]
Female (%)	25 (56.8)
Current smoker (%)	8 (18.2)
Alcohol use (%)	14 (31.8)
Obesity (BMI ≥ 30 Kg/m^2) (%)	8 (18.2)
BMI (median [IQR])	22.88 [20.07, 27.39]
Known HIV-positive status (%)	1 (4.2)
Hypertension	42 (95.5)
Diabetes	9 (22.0)
Source of income	
Depending on others (%)	11 (25.0)
Grant/pension (%)	17 (38.6)
Salary/wage/business (%)	6 (13.6)
Others (%)	10 (22.7)

Table A6. Results of patient cost survey

Variables	Median (IQR) or N (%) unless otherwise indicated
Type of facilities visited	
Public clinic (%)	43 (97.7)
General practitioner (%)	1 (2.3)
Number of clinic visits	
Once	42 (95.5)
Twice	2 (4.5)
Travel time, min (IQR)	30.0 (20.0, 40.0)
Clinic time, min (IQR)	90.0 (43.75, 150.0)
Costs for meal, $ (IQR)	0 (0, 0), five needed to pay for meal, from $0.61 to 1.22
Costs for registration	Only one needed to pay USD 27.5
Costs for examinations or medicine	None required.
Self-reported income loss	Only two reported loss of income-$12.2 and 33.6
Need for an attendant (%)	
Yes	11 (25%)
No	33 (75%)
Need to buy equipment	One bought a blood pressure monitor ($ 18.3)

Table A7. Patient costs for NCD screening

	Average cost per person who was referred (USD)
**Direct costs**	
Equipment	0.42
Transportation cost	1.59
Meal	0.09
Registration	0.49
Total direct costs	2.59
**Indirect costs**	
Self-reported income loss	0.76
Income loss for contacts (minimum wage approach)	3.18
Income loss for attendants (minimum wage approach)	0.79
Total indirect costs using self-reported income loss	0.76
Total indirect costs using minimum wage approach	3.97
**Total costs**	
Total costs using self-reported income	2.93
Total costs using minimum wage approach	5.14

Table A8. Incremental provider costs for NCD screening

Personnel	Hourly wage ($)	Input	Average incremental cost per contact ($)
Research assistants	8.5	Extra 19 minutes per contact, two assistants	5.38
Nurse	13.9	Extra 19 minutes per contact, one nurse	4.41
**Subtotal**			9.79
**Laboratory tests and consumables**	**Unit cost ($)**	**Unit**	**Average incremental cost per contact ($)**
Blood glucose	6.85	One per contact	6.85
HbA1c	23.47	One per contact	23.47
Creatinine Serum	6.85	One per contact	6.85
Total cholesterol	7.82	One per contact	7.82
Sodium Fluoride Glucose Tube	0.27	One per contact	0.27
HbA1c sample collection tube	0.27	One per contact	0.27
Creatinine and T-cho Serum collection tube	0.27	One per contact	0.27
Urine protein dip-stick	0.29	One per contact	0.29
Glove	0.22	One per contact	0.22
Vacutainer needles	0.39	One per contact	0.39
Vacutainer tube holder	0.22	One per contact	0.22
Elastoplast	0.005	One per contact	0.005
Alcohol swabs	0.05	One per contact	0.05
Tourniquet	48.9	One per total number of contacts screened (N = 291)	0.17
Cooler box	48.9	One per total number of contacts screened (N = 291)	0.17
Sharp bins five litres	4.89	One per total number of contacts screened (N = 291)	0.02
Kit construction	1392.67	One per total number of contacts screened (N = 291)	4.79
Transportation cost	1298.78	One per total number of contacts screened (N = 291)	4.46
Blood pressure monitor	45.84	One per total number of contacts screened (N = 291)	0.16
Out-patient consultation	14.55	One per contact referred	3.59
**Sub-total**			60.34
**Programme cost**	**Unit cost ($)**	**Unit**	**Average incremental cost per contact ($)**
Training	427.87	One per total number of contacts screened (N = 291)	1.47
Sub-total			1.47
**Total provider cost per contact screened**			**71.6**

Table A9. Cost-effectiveness of integrated NCD screening within contact investigation-comparison of different targeting strategies

	Primary analysis (All contacts)	Strategy 1 (> Aged 40 years)	Strategy 2 (WHO PEN guidelines[1])	Strategy 3 (WHO PEN guidelines[2])
**10-year CVD risk (%)**	Median: 2.7 (IQR 1.0-5.1)	Median: 5.7 (1.8-12.3)	Median: 2.7 (IQR 1.2-5.2)	Median: 2.7 (IQR 1.2-6.3)
**YLL per 100 persons**	1.2	1.3	1.2	1.4
**YLD per 100 persons**	0.8	0.9	1.0	0.9
**DALYs (discounted) per 100 persons**	1.7	1.8	1.7	1.9
**Incremental cost for screening (USD) per contact screened**	71.6	35.9	38.3	34.5
**Cost for subsequent management (USD) per contact screened**	442.6	332.2	395.8	358.0
**Incremental cost per contact screened**	487.9	341.7	385.2	345.7
**Incremental cost per DALY averted (USD)**	27043.6	20551.3	22040.9	24134.7

[1]CVD risk assessment and blood pressure measurement in adults aged > 40 years, current smokers, or people who are overweight and testing for diabetes in adults aged > 40 years who are overweight.

[2]CVD risk assessment and blood pressure measurement in adults aged > 40 years, current smokers, or people who are overweight and testing for diabetes in adults aged > 40 years who are obese.

Table A10. Probabilistic sensitivity analysis of the cost-effectiveness of integrated NCD screening

	Uncertainty interval of net health benefit
**Primary analysis (All contacts)**	-50.5; -33.3
**Strategy 1 (> Aged 40 years)**	-36.6; -19.9
**Strategy 2 (WHO PEN guidelines[1])**	-43.8; -27.4
**Strategy 3 (WHO PEN guidelines[2])**	-39.4; -24.7

The uncertainty interval represents 2.5th and 97.5th percentile of the distribution of net health benefits.

Figure A1. Probabilistic sensitivity analysis of the cost-effectiveness of integrated NCD screening

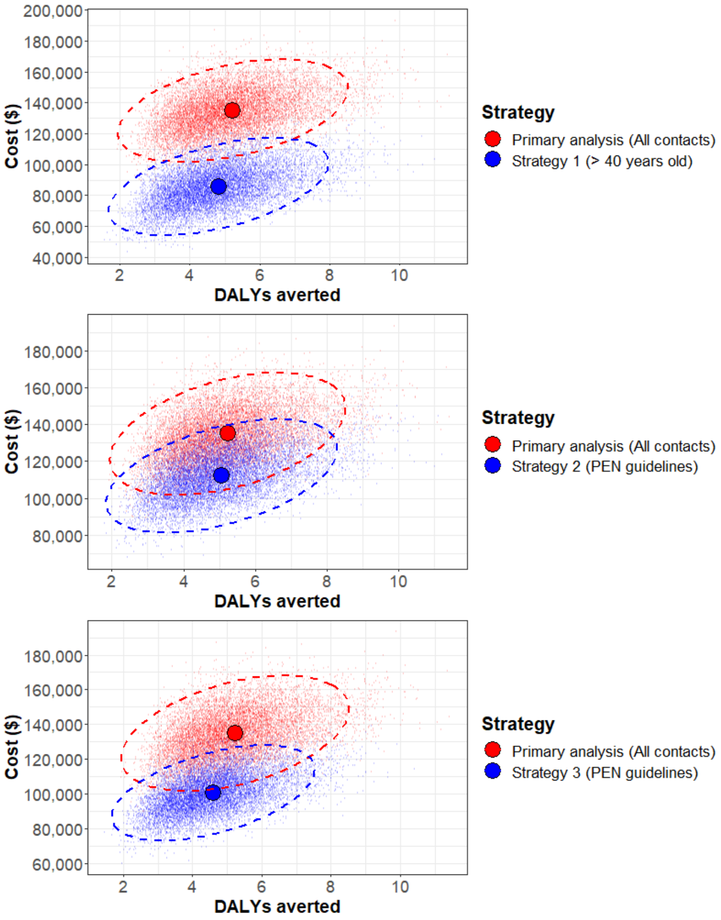

Strategy 2: CVD risk assessment and blood pressure measurement in adults aged > 40 years, current smokers, or people who are overweight and testing for diabetes in adults aged > 40 years who are overweight.
Strategy 3: CVD risk assessment and blood pressure measurement in adults aged > 40 years, current smokers, or people who are overweight and testing for diabetes in adults aged > 40 years who are obese.

Figure A2. Scenario analysis: Probability of being cost-effective by CVD risk in the population

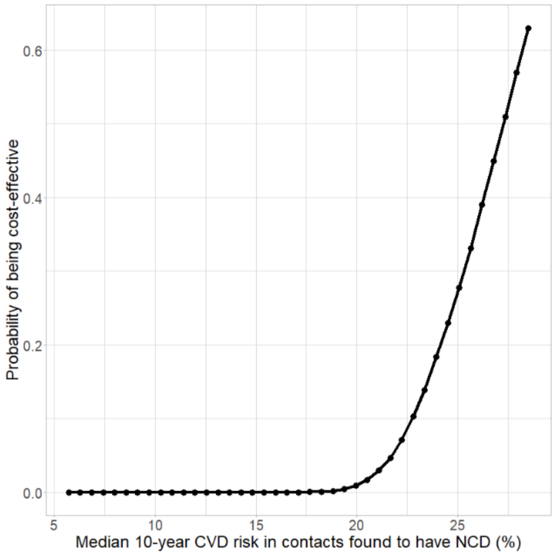

Appendix 2. Questionnaire: The initial cost of care for non-communicable diseases in household contacts who are newly diagnosed with non-communicable disease (NCD)

Question	
1. Have you been hospitalized because of NCD since referral?<br>If yes, go to another questionnaire for individuals who were hospitalized	Yes/No
2. How many clinic visits related to NCD have you had so far since referral (to see the doctor or nurse, have follow- up tests, etc.)?	_____________Times
**Costs required for out-patient visits (repeat 2.1-2.10 for each visit indicated above).**	
2.1 Which of the following types of facilities did you seek care?	**1. Public clinic/hospital**<br>**2. Private clinic/hospital**<br>**3. General practitioner (GP)**<br>**4. Traditional Healer**<br>**5. Pharmacy**<br>**6. Other (specify)**
How long did this clinic visit take, including travel time and waiting time (total turnaround time)?	**Travel time (round trip)**
	**Time at clinic**
2.2 What was the cost of transport (round trip) at the last follow-up medical outpatient visit, including parking, in total for you and any accompanying household member?	
2.3 Did you require accommodation for this visit for staying near the clinic?	Yes/No
If yes, what accommodation cost did you have for this visit, in total, for you and any accompanying household member?	
2.4 Did you have to pay for food as a result of travelling to the hospital/clinic?	Yes/No
If yes, how much did the food cost for this visit, in total, for you and any accompanying household member?	
2.5 What fees did you pay during this medical outpatient visit for <u>registration/consultation</u>?	
2.6 Did you undergo <u>radiography and other imaging</u>?	Yes/ No
If yes, what imaging did you undergo?	Name of the imaging:
What did you pay for this in total?	
2.7 Did you undergo any tests for NCD and others during this medical outpatient visit? They include blood pressure measurement, urine tests, blood tests, peak flow meter, Electrocardiograph and others.	Yes/No
If yes, what did you undergo?	Name of the test:
What did you pay for this in total?	
2.8 Did you require other procedures?	Yes/No
If yes, what were they?	
What fees did you pay for this?	
2.9 Did you lose income because of this visit, for example, because you had to leave from your work?	Yes/No
If yes, how much?	
2.10 Did anyone accompany you to the clinic?	Yes/No

If yes, did that person lose an income during that time?		
2.11 What is his/her monthly income?		
2.12 If you don't want to tell the exact amont, can you tell the category his/her monthly income belong to?	**1 = < R 600**<br>**2 = R 601-1000**<br>**3 = R 1001-2000**<br>**4 = R 2001-4000**<br>**5 = > R 4000**<br>**99 = Don't know**<br>**97 Refused to Answer**	
2.13 Did you have to pay for anything else because of this visit (e.g. child care)?	Yes/No	
If yes, what were they?		
What fees did you pay for this?		
2.14 Did you get reimbursement for this visit from insurance?	Yes/No	
If yes, how much was reimbursed		
**Cost for food**		
3.1 Did you have to change your diet because of NCD, for example, to eat more vegetables and fruits, as recommended by health care staff?	Yes/No	
If yes, how much did you spend on this additional food in the past week approximately?		
**Equipment**		
4. Did you have to buy any special equipment because of your NCD diagnosis (e.g. glucose meter and blood pressure monitor)	Yes/No	
If yes, what equipment did you buy?	Blood pressure monitor	Cost:<br>Name:<br>Maker:
	Blood glucose monitor	Name:<br>Maker:<br>Cost:
	Other	Name:<br>Maker:<br>Cost:

**Medication**				
5.1 List any medications that you were given to treat non-communicable diseases<br>They include medicines to lower blood pressure, blood sugar, or cholesterol.	**Name**	**Dosage (if known)**	**Frequency per day**	**Duration (in days)**

5.2 What fees did you pay for medicines treating NCD, including prescriptions for medicines bought outside the facility?	**None or specific the amount**			
5.3 Were they reimbursed by insurance?	**Yes/No**			
If yes, how much were reimbursed?				
5.4 List any other medicines you were given.	**Name**	**Dosage (if known)**	**Frequency per day**	**Duration (in days)**
5.5 What fees did you pay for other medicines, including prescriptions for medicines bought outside the facility?				
5.6 Were they reimbursed by insurance?	**Yes/No**			
If yes, how much was reimbursed?				
5.7 Were you prescribed insulin?	**Yes/No**			
If yes,	**Dose**	**Frequency**	**Expense**	
Were they reimbursed by insurance?	**Yes/No**			
If yes, how much was reimbursed?				
**Your income**				
What is your individual monthly income?				
If you don't want to tell the exact amont, can you tell the category your monthly income belong to?	**1 = < R 600**<br>**2 = R 601-1000**<br>**3 = R 1001-2000**<br>**4 = R 2001-4000**<br>**5 = > R 4000**<br>**99 = Don't know**<br>**97 Refused to Answer**			

Questionnaire for individuals who were hospitalized because of NCD.

1. How many times were you hospitalized?	______________Times
**Costs required for hospitalization (repeat 2.1-2.10 for each visit indicated above).**	
2.1 Which of the following types of facilities were you hospitalized?	**1. Public hospital**<br>**2. Private hospital**
2.2 Number of days hospitalized	**days**
2.3 Did you have to pay for food during hospitalization?	Yes/No
If yes, how much did the food cost for this hospitalization, in total, for you and any accompanying household member?	
2.4 What fees did you pay during this medical outpatient visit for registration/consultation?	
2.5 Did you undergo radiography and other imaging (e.g. ultrasonography) ?	Yes/ No
If yes, what imaging did you undergo?	Name of the imaging:
What did you pay for this in total?	
2.6 Did you undergo any tests for NCD and others during this hospitalization? They include blood pressure measurement, urine tests, blood tests, peak flow meter, Electrocardiograph and others.	Yes/No
If yes, what did you undergo?	Name of the test:
What did you pay for this in total?	
2.7 Did you require other procedures (e.g. biopsy and surgery)?	Yes/No
If yes, what were they?	
What fees did you pay for this?	
2.8 Were you given any medications during the hospitalization?	Yes/No
What did you pay for this in total?	
2.9 Did anyone accompany you during the hospitalization?	Yes/No
If yes, did that person lose an income during that time?	
What is his/her individual monthly income?	
If you don't want to tell the exact amont, can you tell the category his/her monthly income belong to?	**1 = < R 600**<br>**2 = R 601-1000**<br>**3 = R 1001-2000**<br>**4 = R 2001-4000**<br>**5 = > R 4000**<br>**99 = Don't know**<br>**97 Refused to Answer**
2.10 Did you have to pay for anything else because of this hospitalization (e.g. payment for linen, soap, other services & administrative)?	Yes/No
If yes, what were they?	
What fees did you pay for this?	
2.11 Did you have to pay day charges (e.g. consultation fee) during the hospitalization in addition to costs for the above items?	Yes/No
If yes, how much was it per day?	Per day
2.12 Did you get reimbursement for this visit from insurance?	Yes/No
If yes, how much was reimbursed	
